# Sodium cholate ameliorates nonalcoholic steatohepatitis by activation of FXR signaling

**DOI:** 10.1097/HC9.0000000000000039

**Published:** 2023-01-27

**Authors:** Linyu Pan, Ze Yu, Xiaolin Liang, Jiyou Yao, Yanfang Fu, Xu He, Xiaoling Ren, Jiajia Chen, Xuejuan Li, Minqiang Lu, Tian Lan

**Affiliations:** 1 Guangdong Pharmaceutical University, Guangzhou, Guangdong, China; 2 Department of HBP Surgery II, the Second Affiliated Hospital, School of Medicine, South China University of Technology, Guangzhou, Guangdong, China; 3 Shenzhen Children’s Hospital of China Medical University, Shenzhen, Guangdong, China

## Abstract

Non-alcoholic steatohepatitis (NASH) has become a major cause of liver transplantation and liver-associated death. The gut-liver axis is a potential therapy for NASH. Sodium cholate (SC) is a choleretic drug whose main component is bile acids and has anti-inflammatory, antifibrotic, and hepatoprotective effects. This study aimed to investigate whether SC exerts anti-NASH effects by the gut-liver axis. Mice were fed with an high-fat and high-cholesterol (HFHC) diet for 20 weeks to induce NASH. Mice were daily intragastric administrated with SC since the 11th week after initiation of HFHC feeding. The toxic effects of SC on normal hepatocytes were determined by CCK8 assay. The lipid accumulation in hepatocytes was virtualized by Oil Red O staining. The mRNA levels of genes were determined by real-time quantitative PCR assay. SC alleviated hepatic injury, abnormal cholesterol synthesis, and hepatic steatosis and improved serum lipid profile in NASH mice. In addition, SC decreased HFHC–induced hepatic inflammatory cell infiltration and collagen deposition. The target protein-protein interaction network was established through Cytoscape software, and NR1H4 [farnesoid x receptor (FXR)] was identified as a potential target gene for SC treatment in NASH mice. SC-activated hepatic FXR and inhibited CYP7A1 expression to reduce the levels of bile acid. In addition, high-dose SC attenuated the abnormal expression of cancer markers in NASH mouse liver. Finally, SC significantly increased the expression of FXR and FGF15 in NASH mouse intestine. Taken together, SC ameliorates steatosis, inflammation, and fibrosis in NASH mice by activating hepatic and intestinal FXR signaling so as to suppress the levels of bile acid in NASH mouse liver and intestine.

## INTRODUCTION

Nonalcoholic fatty liver disease (NAFLD), a prevailing chronic liver disease, is often related to metabolic diseases including overweight, diabetes, and hyperlipidemia.^1^ It mainly encompasses the non-progressive nonalcoholic fatty liver (NAFL) and the progressive NASH. NAFL connotes the presence of 5% hepatic steatosis without hepatocyte injury; however, NASH means NAFL is complicated by hepatocellular injury, including with or without fibrosis.^2^ However, to date, no drug has been officially approved for the treatment of NASH.^3^ Therefore, there is an urgent need to discover effective drugs for the treatment of NASH.

Sodium cholate (SC), a cholagogue, is a mixture of sodium taurocholate and sodium glycocholate extracted from bovine bile.^4^ Owing to its multiple pharmacological activities, including anti-inflammatory, antifibrotic, and hepatoprotective effects, SC has been widely used to treat biliary insufficiency, cholecystitis, and cardiovascular disease.^4^ Recent studies have demonstrated that sodium taurocholate reduces serum total cholesterol, low-density lipoprotein cholesterol (LDL-C), and triglyceride (TG) levels in NAFLD rats.^5^ Furthermore, mixing sodium glycocholate with sodium taurocholate attenuates the absorption of bile acids from the portal vein into the liver and blocks their absorption in the enterohepatic circulation, resulting in a decrease in bile acid synthesis.^6^ However, it is not clear whether SC prevents NASH and its underlying mechanism.

The aim of this study was to determine whether SC exerts anti-NASH effects through the gut-liver axis. We used a murine model established by high-fat and high-cholesterol (HFHC) diet feeding, which is characterized by lipid accumulation, inflammation, and fibrosis, and to corroborate the direct effects of SC attenuates hepatocyte injury and lipid accumulation in a palmitic acid (PA)–induced cellular model. We found that SC attenuates bile acid metabolism disorder in NASH mice by activating FXR signaling in the liver and intestine, contributing to the amelioration of steatosis, inflammation, and fibrosis in NASH mice.

## MATERIALS AND METHODS

### Materials and reagents

Reagents and kits were purchased/obtained as follows: Atorvastatin calcium tablets (Pfizer Inc., Dalian, China); Hematoxylin and eosin (H&E) (Biosharp Life Sciences, Hefei, China); Oil Red O (Sigma-Aldrich, St. Louis, USA); Alanine aminotransferase (ALT), Aspartate aminotransferase (AST), Total cholesterol (TC) and TG assay kits (Jiancheng Institute of Biotechnology, Nanjing, China); SYBR Green supermix (Bio-Rad, CA, Berkeley, USA); Anti-rabbit-HRP antibodies (Promega, Madison, WI, USA); Anti-CD68 antibodies (Boster Biological Technology Co, Ltd., Wuhan, China); Anti–toll-like receptor (TLR) (Proteintech Group, Inc., Rosemont, PA, USA); anti-glyceraldehyde-3-phosphate dehydrogenase (GAPDH) antibodies (Boster Biological Technology Co, Ltd., Wuhan, China); and anti-FXR antibodies (Proteintech Group, Inc., Rosemont, PA).

### Preparation of SC

The SC was obtained from the Shanghai Zhonghua Pharmaceutical Co. (Shanghai, China), which belongs to the State Drug Certificate H19999122. SC was dissolved in CMC-Na solution to make a turbid solution of SC and frozen at −20 °C. It was defrosted and injected i.p. at the doses indicated (90 and 180 mg/kg) before daily administration.

### Cell culture and treatment

Human hepatocyte line L02 cell (L02) was obtained from the Chinese Academy of Sciences (Shanghai Institute of Biochemistry and Cell Biology, Shanghai, China). It was cultured in 10% fetal bovine serum (FBS) (Bioind, Israel) and sodium pyruvate solution (Gibco, USA) in RPMI-1640 medium (Thermo, USA) at 37 °C with 5% CO_2_. All cell lines were confirmed to be free of mycoplasma contamination. To emulate the NAFLD model, L02 cells were treated with 0.5 mM PA (Sigma, USA) containing cell culture medium for 24 hours. Bovine serum albumin (0.5%) (BioFroxx, Germany) was used as a control.

### Animal models

Mice were housed in specific pathogen free (SFP) conditions with an ambient temperature of 22–26 °C and humidity of 50–65%, providing 12/12 hours of alternating day and night light and darkness. The NASH was modeled by feeding mice an HFHC (Nantong Trophic; fat, 40%; cholesterol, 2%) for 20 weeks and rendering SC (90 and 180 mg/kg) by gavage after 10 weeks. Mice feeding normal chow (NC) were taken as control. All animal protocols were approved by the Animal Ethics Committee of Guangdong Pharmaceutical University.

### Cell-counting kit-8 assay

The L02 cells viability was measured by cell-counting kit-8 (CCK-8) colorimetric assay (BioSharp, China), according to the manufacturer’s instructions. L02 cells were subcultured in 96-well plates with complete medium and treated with different concentrations of SC for 24 hours. After treatments, CCK8 reagent was added into each well, followed by incubating for another 1.5 hours in a 37 °C incubator with 5% CO_2_. Then, the absorbance was measured by a microplate reader at 450 nm (Bio-Rad, Hercules, CA, USA ), and cell viability was expressed as percentage values, as compared with the control group.

### Lactate dehydrogenase

An lactate dehydrogenase (LDH) cytotoxicity assay kit (Jiancheng Institute of Biotechnology, Nanjing, China) was used to examine the release level of LDH according to the instructions, and the optical density of the samples was measured by a microplate spectrophotometer (Bio-Rad, Hercules, CA, USA) at 450 nm. The level of cytokines was measured using ELISA-based kits, according to the manufacturer’s instructions.

### Cellular Oil Red O staining

L02 cells were treated with 0.5 mM PA, rinsed twice with PBS, fixed with 4% paraformaldehyde for 15 minutes, and then stained with 60% Oil Red O working solution for 5 minutes. After washing three times with deionized water, images were observed under a microscope (Olympus, Tokyo, Japan).

### Glucose and insulin tolerance tests

The glucose tolerance test (GTT) was performed on mice fed with HFHC diet for 16 weeks. One week later, the same mice were performed by the insulin tolerance test (ITT). For GTT, mice were fasted for 16 hours. After measuring the baseline blood glucose level by means of a tail nick using a glucometer, 2 g/kg glucose was administered by means of intragastric injection, and glucose levels were measured 15, 30, 60, and 120 minutes after glucose injection. For ITT, mice fasted for 6 hours were injected i.p. with insulin at 0.5 U/kg and their blood glucose concentrations were determined 15, 30, 45, and 60 minutes after insulin injection.

### Serum assays

Serum TG, TC, high-density lipoprotein (HDL), low-density lipoprotein (LDL), ALT and AST levels were measured by a commercial kit, according to the manufacturers’ instruction (Jiancheng Bioengineering Institute, Nanjing, China).

### Quantitative analysis of hepatic TG and total cholesterol

Hepatic TG and TC were extracted from liver tissues with a mixture of chloroform and methanol. The contents of hepatic TG and TC were measured by a commercial kit (Jiancheng Bioengineering Institute, Nanjing, China) and normalized by liver wet weight.

### Quantitative analysis of hepatic free fatty acids and free cholesterol

Hepatic free fatty acid (FFA) and free cholesterol (FC) were extracted from liver tissues with saline. The contents of hepatic FFA and FC were measured by a commercial kit (Jiancheng Bioengineering Institute, Nanjing, China) and normalized by liver wet weight.

### Histopathology

Liver tissue was fixed overnight in 4% paraformaldehyde solution (4 °C), embedded in paraffin, and then sectioned (4 μm) for hematoxylin and eosin (H&E) staining to visualize liver ballooning, steatosis, and inflammatory cell infiltration. Picrosirius red (PSR, 26357-02; Hede Biotechnology Co., Ltd., Beijing, China) staining was performed to visualize the degree of liver fibrosis. The positive areas were quantified using the Image J software. Histologic images of section tissues were captured with a light microscope (Olympus, Tokyo, Japan).

### NAFLD activity score scores

The NAFLD activity score (NAS) score is a semiquantitative scoring system. The main contents of NAS scores are hepatocyte steatosis, hepatic lobular inflammation, and hepatocyte ballooning. The total NAS score is the sum of these 3 scores: (1) hepatocyte steatosis: 0 (<5%), 1 (5%–33%), 2 (34%–66%), 3 (>66%); (2) lobular inflammation 0 (none), 1 (<2), 2 (2–4), 3 (>4); (3) hepatocyte ballooning: 0 (none); 1 (rare); 2 (common).

### Immunohistochemistry

Liver specimens fixed in 4% paraformaldehyde solution were embedded in paraffin blocks. Liver sections (4 μm thick) were processed using a standard immunostaining protocol. For immunohistochemical analysis, liver sections were separated, rehydrated, and incubated sequentially with primary and secondary antibodies. The area of positive staining was measured in high magnification fields on each slide and quantified using Image J.

### Real-time quantitative PCR

Total RNA of liver tissues was extracted using TRIzol reagent (Thermo, USA), followed by reverse transcription and quantitative real-time PCR (q-PCR). From the extracted mRNA, cDNA was synthesized using the PrimeScript™ RT reagent kit with gDNA Eraser (Takara, Beijing, China). All the primer sequence information was shown in Table 1. Q-PCR assay was performed using the SYBR Green Supermix (Bio-Rad, Hercules, CA, USA). The relative amount of each mRNA was calculated by using the comparative threshold cycle method. Comparative threshold values were normalized to GAPDH.

**TABLE 1:** Primer sequences for real-time PCR

Primer name	Forward primer sequence	Reverse primer sequence
Human		
*GAPDH*	GGAGCGAGATCCCTCCAAAAT	GGCTGTTGTCATACTTCTCATG
*CHREBP*	GCGTTTTGACCAGATGCGAGAC	CGTTGAAGGACTCAAACAGAGGC
*SREBP-1C*	ACTTCTGGAGGCATCGCAAGCA	AGGTTCCAGAGGAGGCTACAAG
*DGAT1*	GCTTCAGCAACTACCGTGGCAT	CCTTCAGGAACAGAGAAACCACC
*CD36*	GTGTGGTGATGTTTGTTGCTTT	CTGGATAAGCAGGTCTCCAACT
*PPAR-α*	CAAGAAGACGGAGTCGGATG	CGTAAAGCCAAAGCTTCCAG
*ATGL*	CCCACTTCAACTCCAAGGACGA	GCAGGTTGTCTGAAATGCCACC
*HSL*	AGCCTTCTGGAACATCACCGAG	TCGGCAGTCAGTGGCATCTCAA
*MGL*	GGCATGGTACTCATTTCGCCTC	GTTTGGCAGCACAAGGTTGAGC
**Mouse**		
*Gapdh*	GTCAAGGCCGAGAATGGGAA	CTCGTGGTTCACACCCATCA
*Scap*	CCGAGCATTCCAACTGGTG	CCATGTTCGGGAAGTAGGCT
*Srebp2*	GCAGCAACGGGACCATTCT	CCCCATGACTAAGTCCTTCAACT
*Hmgcr*	TGTTCACCGGCAACAACAAGA	CCGCGTTATCGTCAGGATGA
*Srebp-1c*	CGACTACATCCGCTTCTTGCAG	CCTCCATAGACACATCTGTGCC
*Dgat1*	CCGTGTTTGCTCTGGCATC	TGACCTTCTTCCCTGTAGAG
*Cd36*	TGAGACTGGGACCATTGGTGAT	CCCAAGTAAGGCCATCTCTACCAT
*Ppar-α*	TATTCGGCTGAAGCTGGTGTAC	CTGGCATTTGTTCCGGTTCT
*Atgl*	GAGGAATGGCCTACTGAACCA	GGCTGCAATTGATCCTCCTCT
*Il-1β*	CCGTGGACCTTCCAGGATGA	GGGAACGTCACACACCAGCA
*Ccl2*	TACAAGAGGATCACCAGCAGC	ACCTTAGGGCAGATGCAGTT
*Ccl5*	TGCTGCTTTGCCTACCTCTC	TCTTCTCTGGGTTGGCACAC
*Fxr*	GCTTGATGTGCTACAAAAGCTG	CGTGGTGATGGTTGAATGTCC
*Shp*	TGGGTCCCAAGGAGTATGC	GCTCCAAGACTTCACACAGTG
*Cyp7a1*	GGGATTGCTGTGGTAGTGAGC	GGTATGGAATCAACCCGTTGTC
*Cyp8b1*	TCCTCAGGGTGGTACAGGAG	GATAGGGGAAGAGAGCCACC
*Tnf-α*	GACGTGGAACTGGCAGAAGAG	TTGGTGGTTTGTGAGTGTGAG
*Afp*	TCACATCCACGAGGAGTGTTG	GCGTGAATTATGCAGAAGCCTA
*Ki67*	CAAGGCGAGCCTCAAGAGATA	TGTGCTGTTCTACATGCCCTG
*Fgf15*	ATGGCGAGAAAGTGGAACGG	GGACCAGCGGAGTACAGGT
*Asbt*	GTCTGTCCCCCAAATGCAACT	CACCCCATAGAAAACATCACCA
*Ostβ*	AGATGCGGCTCCTTGGAATTA	TGGCTGCTTCTTTCGATTTCTG
*Tlr4*	CTGCAATGGATCAAGGACCA	TTATCTGAAGGTGTTGCACATTC
*Tlr2*	CCCATTGCTCTTTCACTGCT	CTTCCTTGGAGAGGCTGATG
*Il6*	AGGAGTGGCTAAGGACCAAGACC	TGCCGAGTAGACCTCATAGTGA

Abbreviations: *Afp*, α-fetoprotein; *Asbt*, apical sodium-dependent bile acid transporter; Atgl, adipose triglyceride lipase; *Ccl2*, C-C motif chemokine ligand 2; *Ccl5*, C-C motif chemokine ligand 5; *Fxr*, farnesoid x receptor; *Ostβ*, organic solute transporter β; *Shp*, small heterodimer partner.

### Western blot analysis

The L02 cells were lysed with ice-cold RIPA lysis buffer (65 mM Tris-HCl pH 7.5, 150 mM NaCl, 1 mM EDTA, 1% Nonidet P-40, 0.5% sodium deoxycholate, and 0.1% SDS) supplemented with a protease inhibitor (Roche, Basel, Switzerland) and a phosphatase inhibitor (Roche, Basel, Switzerland) and centrifuged at 12,000 rpm for 30 minutes at 4 °C. Total proteins (20–40 µg) were electrophoresed on SDS-PAGE gels and transferred to polyvinylidene fluoride membranes (Millipore). Afterwards, the membranes were blocked with 10% nonfat dry milk, followed by incubation with primary and secondary antibodies. Membranes were detected by Clarity Western electrochemiluminescence (ECL) Substrate (Bio-Rad, USA) in conjunction with a chemiluminescence system (New Life Science Products, USA).

### Statistical analysis

All data were statistically analyzed by using GraphPad Prism 8.3.0 (San Diego, California, USA), and the results were expressed as the mean±SD. Differences between the means of the 2 groups were analyzed by a 2-tailed unpaired Student’s t-test and were considered statistically significant when *p*<0.05, while for comparisons between more than 2 groups a one-way ANOVA was performed.

## RESULTS

### SC ameliorates PA-induced hepatocyte injury and lipid accumulation in L02 cells

**FIGURE 1:**
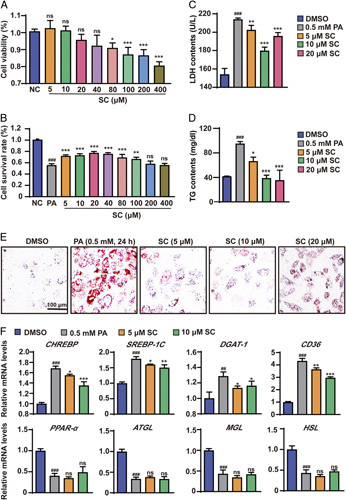
SC reduces hepatocyte injury and lipid accumulation in L02 cells treated with PA. (A) The effect of SC on L02 cell viability. (B) The effect of SC on L02 cell cytotoxicity. (C) Lactate dehydrogenase contents in L02 cells in the indicated groups treated with PA (0.5 mM) for 24 hours. (D) Triglyceride contents in L02 cells in the indicated groups stimulated with PA (0.5 mM) for 24 hours. (E) Oil Red O staining showing the degrees of lipid accumulation in L02 cells treated with DMSO, 5,10, and 20 μM SC in response to PA (0.5 mM) stimulation for 24 hours. Scale bar, 100 μm. (F) Relative mRNA levels of the indicated lipid synthesis (*CHREBP, SREBP-1C*, and *DGAT-1*), lipid uptake genes (*CD36*), and lipid metabolism-related genes (*PPAR-α*, *ATGL*, *MGL*, and *HSL*) in L02 cells treated with PA (0.5 mM). n=3 per group. The Data are presented as the mean±SD. # indicates a significant difference between the DMSO group and the PA (0.5 mM) group (*t* test); *indicates a significant difference between the SC (5 μM)/SC (10 μM)/SC (20 μM) group and the PA (0.5 mM) group (one-way ANOVA). ^##^ *p*<0.01, ^###^ *p*<0.001 versus DMSO group; *p<0.05, **p<0.01, ***p<0.001 versus cells induced by PA. Abbreviations: ATGL, adipose triglyceride lipase; CD36, fatty acid translocase CD36; CHREBP, carbohydrate response element binding protein; DGAT1, diacylglycerol acyltransferase 1; HSL, hormone-sensitive lipase; MGL, monoacylglycerol lipase; NS, no significance; PA, palmitic acid; PPAR-α, peroxisome proliferator-activated receptor-α; SC, sodium cholate; SREBP-1C, sterol regulatory element-binding protein 1C.

We examined the effect of SC on hepatocyte viability using the CCK8 assay, and the results showed that when the SC concentration was higher than 80 μM, the viability of hepatocytes was decreased (Figure 1A). Next, L02 cells were treated with 0.5 mM PA for 24 hours to establish a cell model of NAFLD and simultaneously administered with various concentrations of SC. The results showed that the cell survival rate gradually increased as the SC concentration increased from 5 to 20 μM, whereas the cell survival rate gradually decreased when the SC concentration increased from 40 to 400 μM (Figure 1B). Thus, 5, 10, and 20 μM of SC were selected for the subsequent cell experiments. LDH level in the supernatant of L02 cells was elevated by PA induction, suggesting hepatocyte was damaged by lipid toxicity. However, LDH level in hepatocyte was reduced by SC treatment (Figure 1C). The TG content in L02 cells was induced by PA, whereas reduced by SC treatment (Figure 1D). This finding was further confirmed by Oil Red O staining (Figure 1E), suggesting that SC treatment attenuated TG accumulation in L02 cells induced by PA. Moreover, q-PCR assays showed that SC-diminished PA induced the mRNA levels of lipid synthesis such as carbohydrate response element binding protein (*CHREBP*), sterol regulatory element-binding protein 1C (*SREBP-1C*), and *DGAT-1* and lipid uptake genes such as fatty acid translocase CD36 (*CD36*), whereas did not affect other lipid metabolism-related genes such as peroxisome proliferator-activated receptor-α (*PPAR-α*), adipose triglyceride lipase (*ATGL*), monoacylglycerol lipase (MGL), and hormone-sensitive lipase (*HSL*) (Figure 1F), suggesting that SC reduced lipid synthesis and uptake in L02 cells induced by PA. Collectively, these data demonstrated that SC attenuated hepatocyte injury and lipid accumulation induced by PA in hepatocytes.

### SC attenuates HFHC-induced obesity and insulin resistance in mice

**FIGURE 2:**
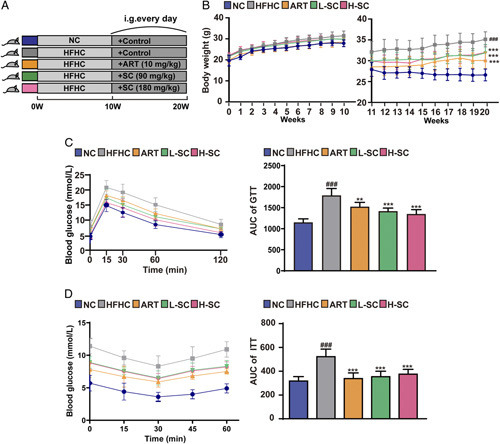
SC alleviates insulin resistance and glucose intolerance in mice fed an HFHC diet. (A) A mouse model of NASH was established schematically to investigate the effects of SC on NASH; we fed mice with this high-calorie diet for 20 weeks and treated them with SC (90 and 180 mg/kg/d) starting at week 11. (B) Body weight in the first 10 weeks and the last 10 weeks of the mice. n=10 per group. (C, D) The GTT and ITT assays were performed to evaluate the insulin sensitivity of the indicated groups of mice treated with NC, HFHC, or SC. n=6—10 per group. Data are represented as means±SD. # indicates a significant difference between the NC group and the HFHC group (*t* test); *indicates a significant difference between the L-SC (Low dose-Sodium Cholate: 90 mg/kg)/H-SC (High dose-Sodium Cholate: 180 mg/kg)/ART group and the HFHC group (one-way ANOVA). ^###^ *p*<0.001 versus NC mice; **p<0.01, ***p<0.001 versus mice fed by HFHC. Abbreviations: ART, atorvastatin; HFHC, high-fat and high-cholesterol; ITT, insulin tolerance test; GTT, glucose tolerance test; NC, normal chow.

To investigate the effects of SC on NASH, we established a NASH mouse model induced by an HFHC diet. Mice were fed with HFHC diet for 20 weeks and treated with SC (90 and 180 mg/kg/d) since the 11th week (Figure 2A). In the 10th week, there were no significant differences in the body weights between the NASH mice and the control mice. At the end of the 20th week, the body weights of the NASH mice were more than those of normal mice, whereas the body weights of the NASH mice treated with atorvastatin (ART) and SC were lower than those of the NASH mice without any treatment (Figure 2B). To assess the effect of SC on insulin resistance in NASH mice, we performed GTT and ITT. After 20 weeks of administration of a high-calorie diet, GTT assay showed that after i.p. injection of glucose, the levels of blood glucose of the mice rapidly reached the peak after 15 minutes, whereas decreased to the baseline after 120 minutes (Figure 2C). The AUC of GTT in NASH mice was larger than that of the normal chow mice; the AUC of GTT in NASH mice with SC treatment was smaller than that of NASH mice without any treatment (Figure 2C). Furthermore, ITT assay showed that after the i.p. injection of insulin, the levels of blood glucose in mice continued to drop in the first 30 minutes, the blood glucose of control mice firstly rebounded after 30 minutes, and the blood glucose of NASH mice started to rebound after 45 minutes (Figure 2D). Moreover, the AUC of ITT in NASH mice was larger than that of the normal chow mice; the AUC of ITT in NASH mice with SC treatment was smaller than that of NASH mice without any treatment (Figure 2D). Taken together, these data suggested that SC treatment improved the rate of glucose metabolism and insulin resistance in NASH mice.

### Effects of SC on hepatic injury and serum lipid profile in HFHC-fed mice

**FIGURE 3:**
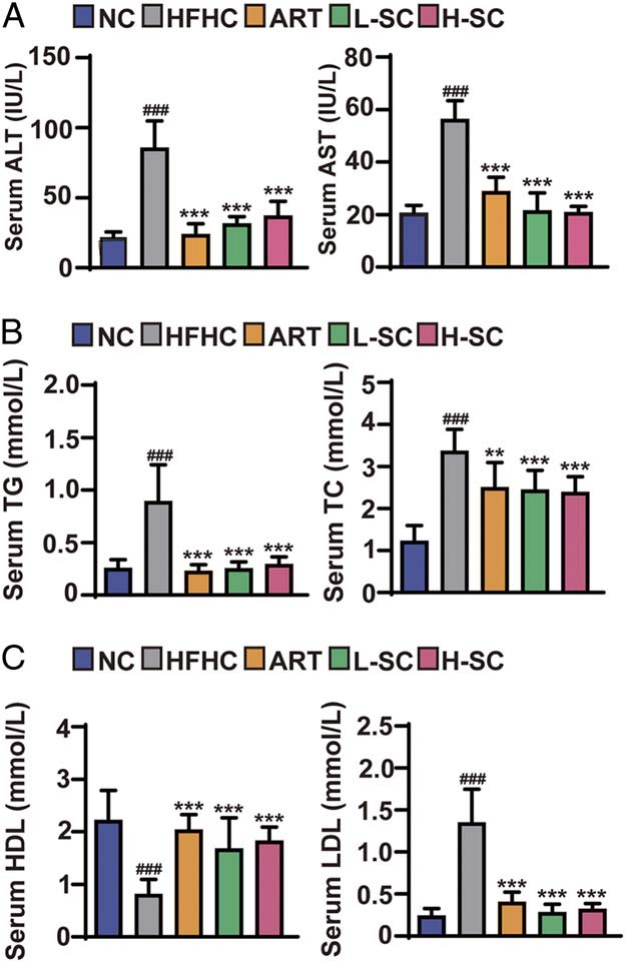
Effects of sodium cholate on serum biochemical parameters in NASH mice. (A) Levels of serum ALT and AST were measured in mice after 20 weeks. n=7–9 per group. (B) Serum lipid (TG and TC) levels. n=7–9 per group. (C) The serum contents of HDL and LDL of mice in the indicated groups. n=7–9 per group. Data are represented as means±SD. #indicates a significant difference between the NC group and the HFHC group (*t* test); *indicates a significant difference between the L-SC (Low dose-Sodium Cholate: 90 mg/kg) H-SC (High dose-Sodium Cholate: 180 mg/kg) group and the HFHC group (one-way ANOVA). ^###^ *p*<0.001 versus NC mice; *p<0.05, **p<0.01, ***p<0.001 versus mice fed by HFHC. Abbreviations: ALT, alanine aminotransferase; AST, aspartate aminotransferase; ART, atorvastatin; HFHC, high-fat and high-cholesterol; TC, total cholesterol; TG, triglyceride.

The above *in vivo* experiments in mice demonstrated the comprehensive protective effect of SC in improving the systemic metabolic stress status in NASH mice. Therefore, we next assayed the levels of serum glutamate-pyruvate ALT and AST, the classical indicators of clinical liver function, and the results showed that SC and ART treatment significantly reduced the abnormal rise of serum transaminases induced by HFHC (Figure 3A). In addition, consistent with the previous results, SC and ART treatment decreased the abnormal elevation of serum TG and TC induced by HFHC, suggesting that SC restored lipid metabolism homeostasis in NASH mice (Figure 3B). Abnormal changes in HDL and LDL are also key serum biochemical indicators in the context of NASH, reflecting the systemic metabolic stress state and the extent of vascular endothelial damage. SC and ART treatment improved the abnormal changes of lipoproteins mentioned above (Figure 3C). These data suggested that SC attenuated hepatic injury and serum lipid profile in NASH mice.

### SC ameliorates hepatic steatosis and injury in HFHC-fed mice

**FIGURE 4:**
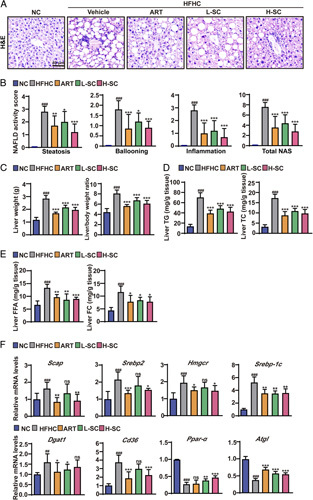
Sodium cholate attenuates hepatic steatosis in mice fed the HFHC diet. (A) Hepatic steatosis was measured by H&E staining. Scale bar, 50 μm. (B) NAFLD activity score in the indicated groups. n=7–10 per group. (C) Liver weight and Liver/body weight ratios of the mice. n=8–10 per group. (D) Hepatic lipid (TG and TC) levels. n=7–8 per group. (E) Hepatic lipid (FFA and FC) levels. n=7–8 per group. (F) Quantitative real-time PCR analysis of the transcript levels of genes related to cholesterol synthesis gene (*Scap, Srebp2* and *Hmgcr*), lipogenic genes (*Srebp-1c* and *Dgat1*), lipid uptake genes (*Cd36*), oxidative phosphorylation gene *(Ppar-α*), and lipolytic gene (*Atgl*). n=7–9 per group. The Data are presented as the mean±SD. #indicates a significant difference between the NC group and the HFHC group (*t* test); *indicates a significant difference between the L-SC (Low dose-Sodium Cholate: 90 mg/kg)/H-SC (High dose-Sodium Cholate: 180 mg/kg)/ART group and the HFHC group (one-way ANOVA). ^##^ *p*<0.01, ^###^ *p*<0.001 versus NC mice; *p<0.05, **p<0.01, ***p<0.001 versus mice fed by HFHC. ns indicates no significance. Abbreviations: ART, atorvastatin; ATGL, adipose triglyceride lipase; CD36, fatty acid translocase CD36; DGAT1, diacylglycerol acyltransferase 1; HMGCR, HMG-CoA reductase; FFA, free fatty acid; FC, free cholesterol; H&E, hematoxylin-eosin; HFHC, high-fat and high-cholesterol; NC, normal chow; PPAR-α, peroxisome proliferator-activated receptor-α; SREBP cleavage-activating protein; SREBP-1C, sterol regulatory element-binding protein 1C; TC, total cholesterol; TG, triglyceride.

H&E staining exhibited significant steatosis and hepatocyte ballooning in the liver of NASH mice. Nevertheless, treatment with SC dramatically reduced these 2 lesions and its effect is similar to that of ART (Figure 4A). On the basis the above pathological staining results, we assessed the liver injury in mice by 3 histological features, namely the degree of steatosis, the number of inflammatory lesions in the liver lobules, and the degree of ballooning of hepatocytes, by NAS score. The results showed that SC treatment significantly improved hepatic steatosis and injury in HFHC-induced NASH mice (Figure 4B). At week 20, the liver weight and the liver/body weight ratio were increased in NASH mice. However, both were decreased in NASH mice treated with ART and SC compared with NASH mice without any treatment (Figure 4C). Hepatic TG and TC accumulation in HFHC-fed mice was significantly decreased by SC treatment (Figure 4D). However, when the liver is chronically exposed to high levels of FC and FFA, it increases the body’s insulin resistance,^7^ causing lipotoxicity and promoting the secretion of inflammatory cytokines, leading to hepatocyte damage and progressive fibrosis during NASH. Therefore, we further assayed these 2 key indicators, FFA and FC, and our data suggest that in a mouse model of NASH, SC and ART treatment significantly reduces the levels of FFA and FC (Figure 4E). To confirm the molecular mechanism of SC for restoring hepatic lipid metabolism homeostasis, we next assayed a representative series of genes for cholesterol and lipid metabolism, including genes for cholesterol and lipid biosynthesis, lipolysis, and uptake, using q-PCR assays. The results showed that the mRNA levels of cholesterol synthesis and lipogenesis genes such as SREBP cleavage-activating protein (*Scap*), *Srebp2*, HMG-CoA Reductase (*Hmgcr*), *Srebp-1c*, and *Dgat1* were increased in HFHC-fed mice, whereas decreased by SC and ART treatment (Figure 4F). In addition, SC and ART treatment decreased the mRNA levels of hepatic lipid uptake genes such as *Cd36* in HFHC-fed mice (Figure 4F). Conversely, the hepatic mRNA levels of *Ppar-α* and *Atgl* were decreased in HFHC-fed mice, whereas increased by SC and ART treatment (Figure 4F). These data suggested that SC attenuated HFHC-induced hepatic steatosis and disorder of hepatic lipid metabolism in mice.

### SC attenuated liver inflammation and progressive fibrosis in HFHC-fed mice

**FIGURE 5:**
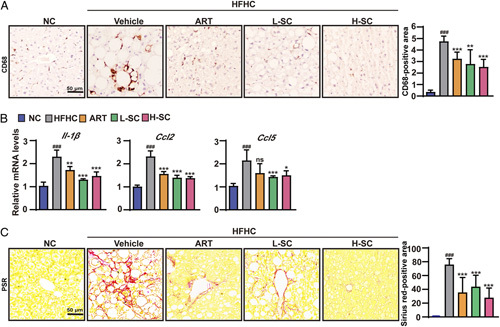
Sodium cholate suppresses hepatic inflammation and fibrosis in mice fed an HFHC diet. (A) Immunohistochemical staining of CD68 in the livers of the indicated mice fed the normal chow or HFHC diet for 20 weeks. n=5 per group. Scale bar, 50 μm. (B) Quantitative real-time PCR analysis of the transcript levels of genes related to inflammation (*Il-1β, Ccl2*, and *Ccl5*). n=5 per group. (C) Representative images showing PSR staining in the livers of the indicated mice fed the normal chow or HFHC diet for 20 weeks. n=5 per group. Scale bar, 50 μm. The data are presented as the mean±SD. #indicates a significant difference between the NC group and the HFHC group (*t* test); *indicates a significant difference between the L-SC (Low dose-Sodium Cholate: 90 mg/kg)/(High dose-Sodium Cholate: 180 mg/kg)/ART group and the HFHC group (one-way ANOVA). ^###^ *p*<0.001 versus NC mice; *p<0.05, **p<0.01, ***p<0.001 versus mice fed by HFHC. Abbreviations: ART, atorvastatin; CCL2, C-C motif chemokine ligand 2; CCL5, C-C motif chemokine ligand 5; CD68, fatty acid translocase CD68; HFHC, high-fat and high-cholesterol; NC, normal chow; NS, no significance; PSR, picrosirius red.

To further investigate the protective effects of SC on NASH *in vivo*, we evaluated the effects of SC on liver inflammation and fibrosis in NASH mice. Immunohistochemical staining of CD68 showed that NASH mice treated with SC-exhibited decreased inflammatory cell infiltration compared with NASH mice without any treatment (Figure 5A). Furthermore, SC treatment significantly decreased the hepatic mRNA levels of inflammatory cytokines such as *Il-1β*, *Ccl2*, and *Ccl5* in NASH mice (Figure 5B). In addition, PSR staining showed that SC treatment significantly reduced the hepatic collagen deposition in NASH mice, thus slowing down the progression of liver fibrosis (Figure 5C). Together, these data suggested that SC attenuated hepatic inflammation and fibrosis in NASH mice under a metabolic stress condition.

### SC activates FXR signaling in NASH mice

**FIGURE 6:**
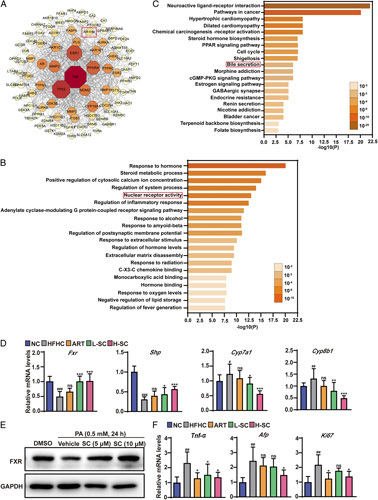
Hepatic farnesoid x receptor (FXR) is required by sodium cholate (SC) treatment in NASH mice. (A) Protein-protein interaction network (PPI) network of SC on NASH. The network contains 97 nodes and 295 edges. Nodes represent proteins and edges represent protein-protein associations. The darker color nodes represent greater degree of freedom (DOF). (B) GO functional annotation of SC on NASH. Bar plot shows the top 20 GO enrichment terms. (C) Kyoto encyclopedia of genes and genomes (KEGG) enrichment analysis of SC on NASH. Bar plot shows the top 20 KEGG pathway terms and corresponding targets. Different colors of the graph represent different signal pathways. The darker color bars in the pathway, the more targets are enriched. (D) Quantitative real-time PCR analysis of the transcript levels of genes related to bile acid metabolism (*Fxr*, *Shp*, *Cyp7a1*, and *Cyp8b1*). n=7 per group. (E) Western blotting of proteins involved in the FXR-mediated signaling in cells. Glyceraldehyde-3-phosphate dehydrogenase served as a loading control. (F) Quantitative real-time PCR analysis of the transcript levels of genes related to cancer markers (*Tnf-α*, *Afp*, and *Ki67*). n=6-7 per group. Data are represented as means±SD. #indicates a significant difference between the NC group and the HFHC group (*t* test); *indicates a significant difference between the L-SC (Low dose-Sodium Cholate: 90 mg/kg)/H-SC (High dose-Sodium Cholate: 180 mg/kg)/ART group and the HFHC group (one-way ANOVA). ^#^ *p*<0.05, ^##^ *p*<0.01, ^###^ *p*<0.001 versus normal chow mice; *p<0.05, **p<0.01, ***p<0.001 versus mice fed by HFHC. Abbreviations: ns, no significance; PPAR, peroxisome proliferator-activated receptor.

To disclose the key target proteins of SC against NASH, protein-protein interaction network consisted of 97 nodes and 295 edges (Figure 6A) was acquired from the gene expression STRING database.^8^ Among the target proteins with degree >5, TP53, TNF-α, PPARA, and NR1H4 (FXR) were reported to involve in multiple biological processes contributed to the NASH progression.^9^ Among the protein-protein interaction network, the cluster containing NR1H4 (FXR) had 25 targets with an average score of 2.48; the cluster containing TNF-α had 20 targets with an average score of 2.05; and the cluster containing PPARA had 23 targets with an average score of 1.62 (the cluster score represents the core density of nodes and topologically adjacent nodes, with higher scores representing more concentrated clusters), suggesting that NR1H4 (FXR) might be a potential direct target gene for SC treatment of NASH (Figure 6A). GO enrichment: GO Ontology enrichment^10^ showed that there were 101 shared targets were enriched with a *p*-value threshold of 0.01. Among the top 20 enrichment results, Response to hormone, Steroid metabolic process, Positive regulation of cytosolic calcium ion concentration and nuclear receptor activity were enriched to a relatively high degree and decreased *p*-values. Thus, it is suggested that nuclear receptor activity might be associated with FXR (Figure 6B). Among 101 outstandingly enriched Kyoto encyclopedia of genes and genomes (KEGG) pathway were obtained from the metascape database,^11^ top 20 ranking KEGG pathway by *p*-value were descripted in Figure 6C, of which the most target proteins of SC were enriched in PPAR signaling pathway, cell cycle, and bile secretion. Considering the important role of bile acids in the process of NASH, we further investigated the genes related to bile acid synthesis in the liver tissue of NASH mice. FXR, as a key gene, mediates bile acid metabolism.^12^ Therefore, to verify the role of SC in bile acid metabolism, we examined genes involved in hepatic bile acid synthesis-related genes. The hepatic mRNA levels of *Shp*, the binding target of *Fxr*, was reduced in HFHC-fed mice, whereas significantly increased by SC and ART treatment (Figure 6D). Conversely, the hepatic mRNA levels of key enzymes of the major bile acid synthesis pathway in liver such as *Cyp7a1* and *Cyp8b1* were increased in HFHC-fed mice, whereas decreased by SC and ART treatment (Figure 6D). To further determine at the protein level whether SC ameliorates NASH by the activation of FXR signaling, we verified the levels of FXR in hepatocytes. SC-activated FXR signaling in hepatocytes subjected to PA stimulation (Figure 6E). The KEGG analysis showed that the cancer pathway was the second most enriched pathway, suggesting high-dose SC treatment significantly decreased the mRNA levels of cancer markers such as *Tnf-α*, α-fetoprotein (*Afp*), and *Ki67* in NASH mice (Figure 6F). Collectively, these data suggested that SC-activated FXR and bile acid synthesis to blunt the pathogenesis of NASH in mice.

### The FXR-FGF15 pathway is required for SC to ameliorate HFHC-induced intestinal inflammation in mice

**FIGURE 7:**
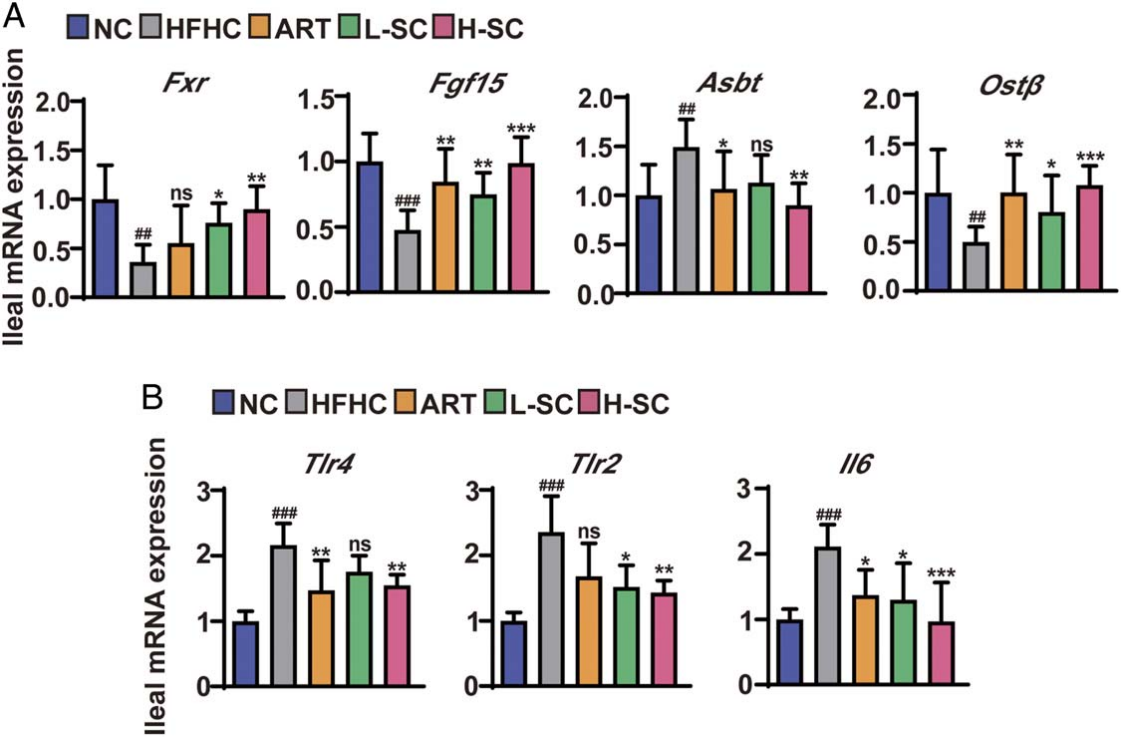
Activation of FXR-FGF15 pathway is responsible for the effect of SC on intestinal inflammation in NASH mice. (A) Quantitative real-time PCR analysis of the transcript levels of genes related to FXR-FGF15 pathway (*Fxr*, *Fgf15*, *Asbt*, and *Ostβ* ). n=8 per group. (B) Quantitative real-time PCR analysis of the transcript levels of genes related to inflammation of the intestinal tract (*Tlr4*, *Tlr2*, and *Il6*). n=8 per group. Data are represented as means±SD. #indicates a significant difference between the NC group and the HFHC group (*t* test); *indicates a significant difference between the L-SC (Low dose-Sodium Cholate: 90 mg/kg)/H-SC (High dose-Sodium Cholate: 180 mg/kg)/ART group and the HFHC group (one-way ANOVA). ^##^ *p*<0.01, ^###^ *p*<0.001 versus NC mice; *p<0.05, **p<0.01, ***p<0.001 versus mice fed by HFHC. Abbreviations: ART, atorvastatin; HFHC, high-fat and high-cholesterol; NC, normal chow; ns, no significance.

Next, q-PCR assays showed that the mRNA levels of *Fxr* and *Fgf15* and bile acid efflux transporter protein [organic solute transporter β, *Ostβ*] in the ileum were significantly decreased in NASH mice, whereas reversed by SC treatment (Figure 7A). In contrast, the mRNA levels of apical sodium-dependent bile acid transporter (*Asbt*) were increased in the ileal tissues in HFHC-fed mice, whereas decreased after SC and ART treatment (Figure 7A). We next assayed the mRNA levels of intestinal inflammatory cytokines in NASH mice. As expected, SC and ART treatment significantly decreased the intestinal mRNA levels of inflammatory cytokines such as *Tlr4, Tlr2*, and *Il-6* in NASH mice (Figure 7B). Collectively, these data suggested that SC treatment protected mice against intestinal inflammation by HFHC induction, suggesting that SC exerts its anti-NASH effects as least in part through intestinal FXR-mediated anti-inflammatory mechanisms.

## DISCUSSION

NAFLD has been the most common chronic liver disease worldwide. There are no approved drugs because of the complexity of the disease and the safety of the drugs.^5^ Thus, it is urgent to explore new drugs to treat NAFLD. SC is a mixture of sodium taurocholate and sodium glycocholate extracted from bovine bile.^13^ In the current study, we demonstrated that SC attenuated lipid accumulation and hepatocyte injury in a PA-induced hepatocytes and inhibited steatosis, inflammation, and fibrosis in NASH mice.

Sterol regulatory element–binding protein (SREBPs) is an essential group of transcription factors regulating lipid synthesis.^14^,^15^ SREBP-1c is involved in hepatic lipid synthesis, and overexpression of SREBP-1c causes hepatic lipid accumulation and insulin resistance.^16^ SREBP2 regulates the expression of genes related to cholesterol synthesis and uptake.^17^ It was found that abnormal expression of SREBP2 would cause disorders of lipid metabolism, especially cholesterol metabolism, which would lead to excessive deposition in adipose tissue and induce NAFLD.^17^ The downstream target gene of SREBP2 is HMG-CoA Reductase (HMGCR).^18^ In NAFLD, an abnormal increase in SREBP2 will promote the synthesis of (HMGCR).^18^ In addition, when the SREBP protein precursor binds to SREBP cleavage-activating protein (SCAP), leading to endoplasmic reticulum stress and exacerbating the imbalance of TG metabolism, which in turn leads to further intracellular lipid accumulation and induces the development of NAFLD.^19^ TG deposition in hepatocytes caused by the disorders of lipid metabolism is the cornerstone of the development of NAFLD.^20^ When the liver is unable to regulate the accumulation of lipids through β-oxidation, FC and FFA form lipotoxic substances, leading to endoplasmic reticulum stress, oxidative stress, and inflammatory vesicle activation.^21^ PPAR-α regulates β-oxidation and the transport of FFA, which plays a crucial role in lipid metabolism.^22^ Liver CHREBP-specific knockdown exacerbates obesity, hepatic steatosis, and insulin resistance in mice and vice versa.^23^ Our results showed that SC treatment significantly decreased the hepatic levels of FFA and FC in NASH mice; meanwhile, the hepatic cholesterol synthesis genes such as *Scap*, *Srebp2*, and *Hmgcr* in NASH mice treated with the mRNA levels of high-dose SC were also reduced; in addition, the mRNA levels of lipogenic genes such as *Srebp-1c* was reduced, whereas the hepatic mRNA levels of *Ppar-α* and *Atgl* were significantly increased in NASH mice treated with SC and ART. These results suggested that SC treatment attenuates hepatic steatosis and lipid metabolism disorders in NASH mice.

The inflammatory process, a hallmark of NASH pathogenesis, is associated with hepatocyte injury and the release of multiple proinflammatory cytokines. The levels of proinflammatory cytokines in the liver, including IL-6, IL-1β, CCL2, and CCL5, are correlated with the severity of NASH.^24^ Macrophages serve a significant role in the pathogenesis of NAFLD. In terms of liver inflammation, the number of CD68-positive cells reflected macrophage recruitment. Macrophage-mediated immune responses are an essential cause of hepatocyte injury during the development of NAFLD.^25^ The accumulation of large amounts of lipids exposes macrophages to prolonged “antigenic” stimuli, inducing the production of IL-6 and causing a sustained inflammatory response.^26^ In addition, inflammation of adipose tissue causes liver injury. In obese mice with severe lipid accumulation, the secretion of IL-1β by adipose tissue macrophages (ATMs) is elevated, which increases the rate of lipolysis of adipocytes and promotes hepatic steatosis.^27^ CCL2 and CCL5 overexpression recruited macrophages that secrete large amounts of inflammatory cytokines and facilitate hepatic steatosis and vice versa, suggesting that the recruitment of macrophages in adipose tissue causes lipid accumulation in mice.^27^ Our results showed that SC suppressed liver and intestinal inflammation in NASH mice by decreasing the mRNA levels of inflammatory cytokines such as *Il-1β, Ccl2*, *Ccl5*, *Tlr4*, *Tlr2*, and *Il6*. Meanwhile, immunohistochemical staining of CD68 demonstrated that treatment with SC reduced inflammatory cell infiltration in livers of NASH mice. These results suggested that SC exerts an anti-inflammatory effect on liver and intestine in NASH mice.

Severe stages of NASH might be accompanied by fibrosis that manifests in the form of excessive deposition of insoluble collagen and extracellular matrix.^28^ HSC is the main source of myofibroblasts, and bone marrow–derived fibroblasts are a potential source of myofibroblasts, which are associated with the pathogenesis of liver fibrosis.^29^ Our results showed that mice fed by HFHC diet developed into liver fibrosis. However, SC reduced the severity of liver fibrosis in the NASH mouse model, thereby hindered the progression of liver fibrosis, suggesting SC exerts an antifibrotic effect on NASH-associated fibrosis.

Bile acids are synthesized from cholesterol in the liver and then transported to the intestine.^30^ They promote the absorption of lipids and fat-soluble vitamins and are important signaling molecules that regulate glucolipid and energy metabolism in the body. Recent studies have shown that disorders of bile acid metabolism are an important cause of the development of NAFLD/NASH.^30^ Dysregulation of bile acid metabolism affects hepatic lipid metabolism, immune microenvironment, and intestinal bacteria. The composition of BAs in the liver and intestine is regulated by BA-metabolizing enzymes and BA transporters, the majority of which are encoded by the FXR target gene that is predominantly expressed in hepatocytes and lower small intestinal epithelial cells, and by the intestinal microbiota.^31^ FXR is a bile acid receptor, which regulates bile acid metabolism by regulating key target genes in all aspects of bile acid metabolism, including bile acid synthesis, metabolism, reabsorption, and transport.^32^ Recent studies have shown that bile acids in the enterohepatic circulation maintain a dynamic balance of bile acids in the body by regulating FXR activity.^33^ When bile acid pooling occurs to the liver, excess bile acids activate FXR and induce increased expression of small hetero dimer partner (SHP).^34^ In the intestine, FXR induced the binding of FGF15/19 to the FGFR4, inhibiting CYP7A1 expression and bile acid synthesis.^35^ In the intestine, conjugated bile acids are first actively reabsorbed into the small intestinal mucosal cells through ASBT in the ileum, and subsequently enter the portal vein by the bile acid efflux transporter OSTα/OSTβ in the basolateral layer.^36^ In addition, many studies have shown that FXR agonists are potential therapeutic drugs for metabolic and inflammatory diseases.^37^,^38^ Obeticholic acid (OCA) is a selective FXR agonist with anticholestatic and hepatoprotective properties,^39^ participating in BA anabolism and enterohepatic circulation, and modulate immune inflammatory and fibrotic.^40^ Since the FXR agonist OCA currently developed for the treatment of NASH was denied marketing approval by the FDA because of its safety and side effects, it is sufficient to demonstrate the critical role that bile acids play in the NASH disease process. Our results showed that SC activates hepatic FXR signaling and induces the expression of downstream SHP, inhibiting the rate-limiting enzyme for bile acid synthesis (CYP7A1), leading to reduced bile acid synthesis in the liver. In addition, SC significantly increased the expression of FXR and FGF15 in NASH mouse intestine. Finally, SC activation of intestinal FXR reduced intestinal bile acid levels by inhibiting the expression of ASBT transporter to bile acid reabsorption, and inducing OSTβ transporter expression to promote bile acid efflux. KEGG analysis showed that the cancer pathway was the second most enriched pathway, so we assayed several cancer markers. Our results suggested that high-dose SC attenuates the abnormal expression of cancer markers such as *Tnf-α*, *Afp*, and *Ki67* in NASH mouse liver. Finally, SC activated FXR signaling in hepatocytes subjected to PA stimulation. Taken together, the above results indicated that SC might activate both hepatic and intestinal FXR expression and regulate bile acid synthesis to impede the pathogenesis of NASH.

**FIGURE 8:**
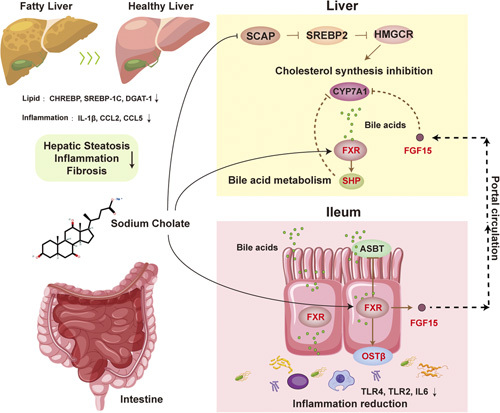
Potential mechanism by which SC attenuated hepatic steatosis, inflammation, fibrosis, and intestinal inflammation in NASH mice. SC reduced the expression of *Srebp-1c* and *Dgat1* in NASH mouse liver, thereby alleviating hepatic steatosis and lipid metabolism disorders. SC suppressed liver and intestinal inflammation in NASH mice by decreasing the mRNA levels of inflammatory cytokines such as *Il-1β, Ccl2, Ccl5, Tlr4, Tlr2* and *Il6*. The protective mechanism of SC not only attributes to the downregulation of *Scap, Srebp2* and *Hmgcr* by SC, inhibiting the synthesis of cholesterol in the NASH mouse liver. More importantly, SC activates hepatic FXR signaling and induces the expression of downstream SHP, inhibiting CYP7A1 expression, leading to reduced bile acid synthesis in the liver. Meanwhile, SC activates intestinal FXR and induces the expression of FGF15, followed by the secretion of FGF15 into the liver and inhibition of CYP7A1 expression in liver and decreased hepatic bile acid synthesis. Furthermore, SC activation of intestinal FXR reduced intestinal bile acid levels by inhibiting the expression of ASBT transporter to bile acid reabsorption, and inducing OSTβ transporter expression to promote bile acid efflux. In conclusion, SC attenuates bile acid metabolism disorder in NASH mice by activating FXR signaling in the liver and intestine, contributing to the amelioration of steatosis, inflammation, and fibrosis in NASH mice. Abbreviations: ASBT, apical sodium-dependent bile acid transporter; CHREBP, carbohydrate response element binding protein; FXR, farnesoid x receptor; HMGCR, HMG-CoA reductase; SREBP cleavage-activating protein; SREBP2, sterol regulatory element-binding protein 2; TLR, toll-like receptor.

Collectively, our current results demonstrated that SC attenuates bile acid metabolism disorder in NASH mice by activating FXR signaling in the liver and intestine, contributing to the amelioration of steatosis, inflammation, and fibrosis in NASH mice. Therefore, our findings will provide insight into the development of clinical treatment for NASH (Figure 8).

## References

[1] Loomba R Friedman SL Shulman GI. Mechanisms and disease consequences of nonalcoholic fatty liver disease. Cell. 2021;184:2537–64. 33989548 10.1016/j.cell.2021.04.015 PMC12168897

[2] Turkish AR. Nonalcoholic fatty liver disease: emerging mechanisms and consequences. Curr Opin Clin Nutr Metab Care. 2008;11:128–33. 18301087 10.1097/MCO.0b013e3282f44bf4

[3] Chalasani N Younossi Z Lavine JE Charlton M Cusi K Rinella M. The diagnosis and management of nonalcoholic fatty liver disease: practice guidance from the American Association for the Study of Liver Diseases. Hepatology. 2018;67:328–57. 28714183 10.1002/hep.29367

[4] Li Y Zhu C. Mechanism of hepatic targeting via oral administration of DSPE-PEG-cholic acid-modified nanoliposomes. Int J Nanomedicine. 2017;12:1673–84. 28280334 10.2147/IJN.S125047 PMC5339015

[5] Liu XJ Liu C Zhu LY Fan CL Niu C Liu XP. Hepalatide ameliorated progression of nonalcoholic steatohepatitis in mice. Biomed Pharmacother. 2020;126:110053. 32200254 10.1016/j.biopha.2020.110053

[6] Fan QY Zhang YT Hou XF Li Z Zhang K Shao Q. Improved oral bioavailability of notoginsenoside R1 with sodium glycocholate-mediated liposomes: Preparation by supercritical fluid technology and evaluation in vitro and in vivo. Int J Pharm. 2018;552:360–70. 30292894 10.1016/j.ijpharm.2018.10.005

[7] Cusi K. Role of obesity and lipotoxicity in the development of nonalcoholic steatohepatitis: pathophysiology and clinical implications. Gastroenterology. 2012;142:711–25 e716. 22326434 10.1053/j.gastro.2012.02.003

[8] Subuddhi U Mishra AK. Micellization of bile salts in aqueous medium: a fluorescence study. Colloids Surf B Biointerfaces. 2007;57:102–7. 17336505 10.1016/j.colsurfb.2007.01.009

[9] Hill EC O’Donnell L. Factors associated with low bone density in a juvenile mortality sample. FASEB J. 2022;36(suppl 1). doi: 10.1096/fasebj.2022.36.S1.R2403

[10] Jiang ZY Zhou Y Zhou L Li SW Wang BM. Identification ofkey genes and immune infiltrate in nonalcoholic steatohepatitis: a bioinformatic analysis. Biomed Res Int. 2021:7561645. doi:10.1155/2021/7561645 34552988 PMC8452393

[11] Kanehisa M Furumichi M Tanabe M Sato Y Morishima K. KEGG: new perspectives on genomes, pathways, diseases and drugs. Nucleic Acids Res. 2017;45:D353–D361. 27899662 10.1093/nar/gkw1092 PMC5210567

[12] Jiang L Zhang H Xiao D Wei H Chen Y. Farnesoid X receptor (FXR): structures and ligands. Comput Struct Biotechnol J. 2021;19:2148–59. 33995909 10.1016/j.csbj.2021.04.029 PMC8091178

[13] Kwak K Yu B Mouli SK Larson AC Kim DH. Sodium cholate bile acid-stabilized ferumoxytol-doxorubicin-lipiodol emulsion for transcatheter arterial chemoembolization of hepatocellular carcinoma. J Vasc Interv Radiol. 2020;31:1697–705 e1693. 32773247 10.1016/j.jvir.2020.01.026 PMC7541531

[14] DeBose-Boyd RA Ye J. SREBPs in lipid metabolism, insulin signaling, and beyond. Trends Biochem Sci. 2018;43:358–68. 29500098 10.1016/j.tibs.2018.01.005 PMC5923433

[15] Zhang X Coker OO Chu ES Fu K Lau HCH Wang YX. Dietary cholesterol drives fatty liver-associated liver cancer by modulating gut microbiota and metabolites. Gut. 2021;70:761–74. 32694178 10.1136/gutjnl-2019-319664 PMC7948195

[16] Horton JD Shimomura I Ikemoto S Bashmakov Y Hammer RE. Overexpression of sterol regulatory element-binding protein-1a in mouse adipose tissue produces adipocyte hypertrophy, increased fatty acid secretion, and fatty liver. J Biol Chem. 2003;278:36652–60. 12855691 10.1074/jbc.M306540200

[17] Xue L Qi H Zhang H Ding L Huang Q Zhao D. Targeting SREBP-2-regulated mevalonate metabolism for cancer therapy. Front Oncol. 2020;10:1510. 32974183 10.3389/fonc.2020.01510 PMC7472741

[18] Zhang YY Fu ZY Wei J Qi W Baituola G Luo J. A LIMA1 variant promotes low plasma LDL cholesterol and decreases intestinal cholesterol absorption. Science. 2018;360:1087–92. 29880681 10.1126/science.aao6575

[19] Chu BB Liao YC Qi W Xie C Du X Wang J. Cholesterol transport through lysosome-peroxisome membrane contacts. Cell. 2021;184:289. 33417863 10.1016/j.cell.2020.12.023

[20] Friedman SL Neuschwander-Tetri BA Rinella M Sanyal AJ. Mechanisms of NAFLD development and therapeutic strategies. Nat Med. 2018;24:908–22. 29967350 10.1038/s41591-018-0104-9 PMC6553468

[21] Kwon EY Jung UJ Park T Yun JW Choi MS. Luteolin attenuates hepatic steatosis and insulin resistance through the interplay between the liver and adipose tissue in mice with diet-induced obesity. Diabetes. 2015;64:1658–69. 25524918 10.2337/db14-0631

[22] Xu J Xiao G Trujillo C Chang V Blanco L Joseph SB. Peroxisome proliferator-activated receptor alpha (PPARalpha) influences substrate utilization for hepatic glucose production. J Biol Chem. 2002;277:50237–44. 12176975 10.1074/jbc.M201208200

[23] Iizuka K Takao K Yabe D. ChREBP-mediated regulation of lipid metabolism: involvement of the gut microbiota, liver, and adipose tissue. Front Endocrinol (Lausanne). 2020;11:587189. 33343508 10.3389/fendo.2020.587189 PMC7744659

[24] Doege H Baillie RA Ortegon AM Tsang B Wu Q Punreddy S. Targeted deletion of FATP5 reveals multiple functions in liver metabolism: alterations in hepatic lipid homeostasis. Gastroenterology. 2006;130:1245–58. 16618416 10.1053/j.gastro.2006.02.006

[25] Zhan YT An W. Roles of liver innate immune cells in nonalcoholic fatty liver disease. World J Gastroenterol. 2010;16:4652–60. 20872965 10.3748/wjg.v16.i37.4652 PMC2951515

[26] Pan X Chen B Liu W Li Y Hu Z Lin X. Circulating iron levels interaction with central obesity on the risk of nonalcoholic fatty liver disease: a case-control study in Southeast China. Ann Nutr Metab. 2019;74:207–14. 30870854 10.1159/000497228

[27] Raghu H Lepus CM Wang Q Wong HH Lingampalli N Oliviero F. CCL2/CCR2, but not CCL5/CCR5, mediates monocyte recruitment, inflammation and cartilage destruction in osteoarthritis. Ann Rheum Dis. 2017;76:914–22. 27965260 10.1136/annrheumdis-2016-210426 PMC5834918

[28] Kisseleva T Uchinami H Feirt N Quintana-Bustamante O Segovia JC Schwabe RF. Bone marrow-derived fibrocytes participate in pathogenesis of liver fibrosis. J Hepatol. 2006;45:429–38. 16846660 10.1016/j.jhep.2006.04.014

[29] Sysa P Potter JJ Liu X Mezey E. Transforming growth factor-beta1 up-regulation of human alpha(1)(I) collagen is mediated by Sp1 and Smad2 transacting factors. DNA Cell Biol. 2009;28:425–34. 19558215 10.1089/dna.2009.0884 PMC2858396

[30] Russell DW. Fifty years of advances in bile acid synthesis and metabolism. J Lipid Res. 2009;50(Suppl):S120–125. 18815433 10.1194/jlr.R800026-JLR200 PMC2674696

[31] Caussy C Hsu C Singh S Bassirian S Kolar J Faulkner C. Serum bile acid patterns are associated with the presence of NAFLD in twins, and dose-dependent changes with increase in fibrosis stage in patients with biopsy-proven NAFLD. Aliment Pharmacol Ther. 2019;49:183–93. 30506692 10.1111/apt.15035 PMC6319963

[32] Dawson PA. Hepatic bile acid uptake in humans and mice: multiple pathways and expanding potential role for gut-liver signaling. Hepatology. 2017;66:1384–6. 28646543 10.1002/hep.29325 PMC5650520

[33] Sun L Cai J Gonzalez FJ. The role of farnesoid X receptor in metabolic diseases, and gastrointestinal and liver cancer. Nat Rev Gastroenterol Hepatol. 2021;18:335–47. 33568795 10.1038/s41575-020-00404-2

[34] del Castillo-Olivares A Campos JA Pandak WM Gil G. The role of alpha1-fetoprotein transcription factor/LRH-1 in bile acid biosynthesis: a known nuclear receptor activator that can act as a suppressor of bile acid biosynthesis. J Biol Chem. 2004;279:16813–21. 14766742 10.1074/jbc.M400646200

[35] Ding L Yang L Wang Z Huang W. Bile acid nuclear receptor FXR and digestive system diseases. Acta Pharm Sin B. 2015;5:135–44. 26579439 10.1016/j.apsb.2015.01.004 PMC4629217

[36] Lin BC Wang M Blackmore C Desnoyers LR. Liver-specific activities of FGF19 require Klotho beta. J Biol Chem. 2007;282:27277–84. 17627937 10.1074/jbc.M704244200

[37] Balasubramaniyan N Luo Y Sun AQ Suchy FJ. SUMOylation of the farnesoid X receptor (FXR) regulates the expression of FXR target genes. J Biol Chem. 2013;288:13850–62. 23546875 10.1074/jbc.M112.443937 PMC3650421

[38] Wang M Liu F Yao Y Zhang Q Lu Z Zhang R. Network pharmacology-based mechanism prediction and pharmacological validation of Xiaoyan Lidan formula on attenuating alpha-naphthylisothiocyanate induced cholestatic hepatic injury in rats. J Ethnopharmacol. 2021;270:113816. 33444723 10.1016/j.jep.2021.113816

[39] Pellicciari R Fiorucci S Camaioni E Clerici C Costantino G Maloney PR. 6alpha-ethyl-chenodeoxycholic acid (6-ECDCA), a potent and selective FXR agonist endowed with anticholestatic activity. J Med Chem. 2002;45:3569–72. 12166927 10.1021/jm025529g

[40] Sun L Cai J Gonzalez FJ. The role of farnesoid X receptor in metabolic diseases, and gastrointestinal and liver cancer. Nat Rev Gastroenterol Hepatol. 2021;18:335–47. 33568795 10.1038/s41575-020-00404-2

